# A Social Justice Approach to Assistive Technology and Well-Being of People With Visual Disabilities in Low- and Middle-Income Countries: Qualitative Narrative Study

**DOI:** 10.2196/72306

**Published:** 2026-04-08

**Authors:** Luisa Maria Ortiz-Escobar, Mario Andres Chavarria, Samia Hurst-Majno, Oscar Ivan Campo Salazar, Celia Escobar-Hurtado, Michael Ashley Stein, Minerva Rivas Velarde

**Affiliations:** 1 Grupo de Investigación en Ingeniería Biomédica, GBIO, Universidad Autónoma de Occidente, Santiago de Cali, Colombia; 2 Institute of Ethics, History, and Humanities, University of Geneva, Geneva, Switzerland; 3 Grupo de Investigaciones en Biomédica, Universidad Autónoma de Occidente, Santiago de Cali, Colombia; 4 EssentialTech Centre, École Polytechnique Fédérale de Lausanne, Lausanne, Switzerland; 5 School of Health Science, HES-SO Genève, Avenue de Champel 47 1206 Genève, Geneva, Switzerland, 41 225585060; 6 Escuela de Rehabilitación Humana, Facultad de Salud, Universidad del Valle, Santiago de Cali, Colombia; 7 Harvard Law School Project on Disability, Harvard University, Cambridge, MA, United States; 8 Faculty of Law Centre for Human Rights, University of Pretoria, Pretoria, South Africa

**Keywords:** assistive technology, persons with disabilities, visual disabilities, user-centered design, capabilities approach, disadvantage theory, LMIC, low- and middle-income countries

## Abstract

**Background:**

The United Nations’ third Sustainable Development Goal emphasizes ensuring healthy lives and promoting well-being (WB) for all, which requires effective assistive technology (AT) for persons with disabilities. In low- and middle-income countries (LMICs), however, AT remains largely inaccessible, and high abandonment rates indicate that many existing solutions fail to meet users’ needs. To improve AT design and effectiveness, a deeper understanding of users’ lived experiences and the ways AT influences WB is essential.

**Objective:**

This study aimed to explore how technology creates opportunities or barriers in the daily lives of persons with visual disabilities in LMICs and how it affects their WB.

**Methods:**

We conducted a qualitative narrative study guided by deductive qualitative analysis, using the capability approach (CA) and disadvantage theory (DT) as theoretical frameworks. Nineteen adults with visual disabilities from Cali, Colombia, participated in in-depth, semistructured interviews. A focus group (n=5) deepened the exploration of shared experiences. Data analysis followed three stages: (1) deductive coding using Nussbaum list of central capabilities and key CA constructs (functionings, conversion factors, and agency); (2) recoding through DT concepts (insecure functioning, corrosive disadvantages, and fertile functionings); and (3) inductive analysis to capture emergent sociocultural themes.

**Results:**

AT shaped both opportunities and constraints in participants’ lives. While functionings such as employment, mobility, and affiliation were highly valued, they often remained insecure due to systemic barriers. Corrosive disadvantages—such as unemployment, exclusion, and limited spatial autonomy—undermined multiple capabilities simultaneously. Conversely, fertile functionings such as equitable employment, adaptive sports, and access to well-designed AT supported agency and resilience. The inductive analysis revealed 3 interconnected themes: the aspiration to explore and expand movement, the desire to appear attractive, and the adoption of nonconfrontational strategies to maintain social harmony. These findings highlight how emotional, aesthetic, and cultural dimensions shape the experience and meaning of AT.

**Conclusions:**

While AT research in LMICs often emphasizes availability, it rarely addresses how social norms, structural violence, and fear affect meaningful use. The combined CA and DT lens reveals that AT can either enable or constrain WB depending on how it aligns with users’ lived contexts. Designing for fertile functionings—those that support agency, safety, and resilience—is essential. Participatory, context-sensitive design must prioritize not only functionality, but also aesthetic dignity, cultural relevance, and emotional security. Including the voices—and silences—of persons with disabilities in the Global South is crucial for transforming AT from a mere tool into a catalyst for real freedom and WB.

## Introduction

### Background

The incidence and prevalence of visual impairment worldwide have exceeded World Health Organization (WHO) projections for the second decade of the current century [1,2]. In low- and middle-income countries (LMICs), the rate of persons with severe visual impairments is approximately 2 to 3 times higher than in high-income countries [3].

Visual impairment significantly constrains individual autonomy and social participation, with far-reaching consequences for quality of life [4]. Assistive technology (AT) has emerged as a critical means of mitigating these effects by promoting independence and enabling greater inclusion.

Beyond its practical benefits, AT is increasingly recognized as instrumental in advancing the rights enshrined in the United Nations Convention on the Rights of Persons with Disabilities [5], as well as in supporting broader global commitments such as Sustainable Development Goal 3: Good Health and Well-Being [6].

Thus, AT contributes to overall well-being (WB) by enhancing autonomy, supporting participation in education, employment, and community life, and reinforcing psychosocial WB through increased agency and participation [7,8].

Nevertheless, despite its benefits, access to AT remains severely constrained—particularly in LMICs [9]. This lack of access is further exacerbated by high abandonment rates, which often result from inadequate alignment between technologies and the lived realities of users [10-12]. The literature increasingly points to the need for participatory, person-centered approaches that actively engage persons with disabilities in the design and delivery of AT solutions [13], ensuring that such interventions are both contextually relevant and sustainable.

Although AT development increasingly claims to adopt user-centered design [14], studies have identified a persistent gap between rhetoric and practice. Users are often consulted only to refine existing designs, rather than to inform fundamental decisions based on their needs and experiences [15,16].

To respond to this gap and advance a genuinely person-centered approach, we initiated a study to inform the development of AT for the navigation of persons with visual disabilities in LMICs. This paper presents the results of the first phase, a qualitative inquiry into users’ lived experiences, aspirations, and interactions with technology.

While AT research in LMICs often focuses on availability, it neglects how structural and social contexts shape meaningful use. The capability approach (CA) addresses this by emphasizing individuals’ real freedoms to achieve valued functionings, not just access to resources [17-19]. However, CA has been critiqued for undertheorizing how power, fear, and structural violence constrain agency [20]. Disadvantage theory (DT) complements CA by introducing concepts such as corrosive disadvantage, enabling deeper analysis of how AT may worsen exclusion under adverse conditions [21]. This combined CA and DT lens highlights how AT can both enable and limit WB in LMICs.

### Approaches to Disability, WB, AT, and Justice

#### The Capabilities Approach

The CA evaluates human development through the lens of equity, emphasizing human flourishing as the ultimate goal. It focuses on the life individuals are able to lead, based on their “capabilities” (practical opportunities) and “functionings” (achievements) [22].

Building on this foundation, this approach recognizes the concept of agency, which is the ability to pursue valued goals. To achieve these goals, individuals need access to necessary resources and “conversion factors,” which affect how resources are transformed into functionings [17].

This theoretical lens has gained traction in disability studies over the past 2 decades, where it emphasizes individual agency and enables a nuanced examination of resources and conversion factors. In this context, disability is framed in terms of the deprivation or restriction of capabilities and functionings [23-26].

For this study, we chose the CA to analyze the experiences of persons with visual disabilities in a middle-income country, namely Colombia. This framework sheds light on what people value in their lives, guides researchers in identifying daily opportunities and barriers, and aids in evaluating how AT influences the pursuit of a “good life.”

Recent literature situates the role of technology within this framework [27,28].

**Figure 1.**
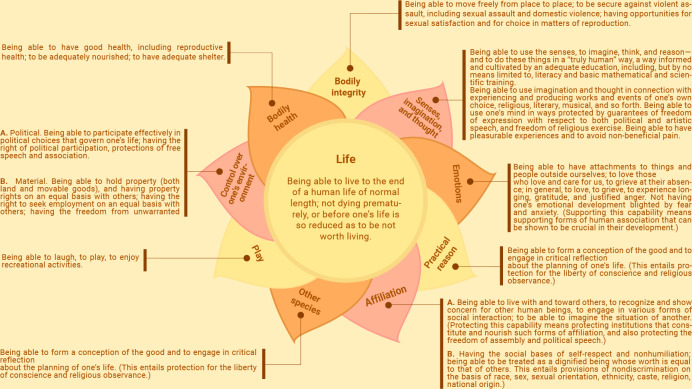
Diagram of the 10 central capabilities proposed by Nussbaum and used as a framework for deductive analysis of participant narratives about technology and well-being.

Nussbaum, who has made valuable contributions to the approach, proposed an open list of 10 capabilities, which she argues are central to the realization of self-determined, meaningful, and fulfilling lives [18,29]. This list (Figure 1) has been effectively applied in various contexts, including assessing dignity, monitoring human rights, and guiding social policy to reduce disadvantage [30].

Disability research focused on understanding quality of life, living conditions, and WB in LMICs is scarce and predominantly quantitative, limiting insights into the lived experiences of disability and personal ideas of flourishing [31,32]. Research on AT within the CA for persons with disabilities is even rarer. This field could benefit from studies like this one, which aim to provide valuable information for developing AT to narrow the capabilities gap. Understanding the lived experience of disability and personal definitions of a good life can guide AT designers in making meaningful and desirable assistive devices (ADs).

#### Disadvantage Theory

Wolff and De-Shalit [21] developed a theory aimed at achieving equity and social justice by addressing disadvantage. Drawing from Sen CA and Nussbaum contributions, they define disadvantage as a “lack of genuine opportunities for secure functionings” [21].

These authors underscore the plural origins of disadvantages and the necessity of indexing them. They diverge from Sen and Nussbaum by using the terms genuine opportunities and secure functionings instead of capabilities and functionings. While Sen emphasizes freedom to achieve capabilities, Nussbaum defends a universal list of central capabilities, Wolff and De-Shalit focus on how disadvantage is experienced and addressed in real-world contexts. Their terminology reflects a concern with the stability of achieved functionings and the substantive nature of opportunity, aiming to make the framework more actionable for policy design.

They define a disadvantaged person as one whose functionings are involuntarily insecure. Another key concept is “corrosive disadvantage,” where one disadvantage negatively impacts another functioning, making it insecure. To counteract this, the authors suggest identifying and addressing corrosive disadvantages while also seeking “fertile functionings,” which positively impact and secure other functionings [21].

Persons with disabilities face multiple disadvantages, such as “reduced health, life span, opportunities for fulfilling work, social networks, control over their lives, leisure activities, and living standards” [21,33]. Given the likelihood of facing corrosive disadvantages, it is crucial to prioritize the capabilities and functionings of persons with disabilities to decluster their disadvantages.

### WB, Visual Disability, and AT

Sen [34] addresses WB from an individual perspective, asserting that it should be evaluated based on the achievement of functionings that each person values for a good life. He states, “functionings are seen as central to the nature of WB, even though the sources of WB could easily be external to the person.” This aligns with the current notion of subjective WB, “a person feeling and thinking his or her life is desirable regardless of how others see it” [35], which is used by organizations and countries to monitor compliance with Sustainable Development Goal 3 [36,37].

Most data on this subject come from high-income countries, showing that persons with disabilities generally have lower WB than those without disabilities, often linked to poverty, educational status, unemployment, and social exclusion [38-41]. A recent study including data from LMICs confirmed this trend, attributing lower WB in persons with disabilities to “differences in living conditions and life experiences in relation to household wealth, level of education, partnership status, and exposure to violence and discrimination in the previous year” [42].

In the context of visual disabilities, common challenges include social isolation, limited mobility, low education, unemployment, stigmatization, and injury risks [43].

ATs are seen as tools to overcome these limitations and improve WB. Despite their potential benefits, not all persons with visual disabilities adopt AT, particularly mobility aids, due to concerns about stigmatization and inadequate obstacle detection [44]. This reluctance indicates that AT does not always enhance WB, underscoring the need to understand user interactions with AT to develop solutions that are more desirable and supportive of users’ WB.

### Aims of This Study

We explored the role of technology in the lives of persons with visual disabilities in LMICs and aimed to answer the following questions: (1) How can AT create opportunities or barriers in daily life? (2) How does technology affect WB?

### Context of Fieldwork

Lived experience, disability, capabilities, and functionings should be understood within their sociocultural context [32,45]. The cultural perception of disability significantly influences both coping strategies and the willingness to use AT. These factors cannot be separated from the historical and political contexts that have shaped the experiences of a particular group [46].

This study took place in Colombia, a country affected by internal armed conflict for nearly 6 decades. The “Comisión de la Verdad (Truth Commission)” reported 8,775,884 victims of human rights violations in the conflict [47].

In 2016, a peace agreement was signed between the Colombian government and the FARC-EP (Revolutionary Armed Forces of Colombia). The historical memory construction process, a process of reconstructing and interpreting the past to dignify victims, clarify the truth about human rights violations, and promote reconciliation in the context of Colombia’s armed conflict, highlighted patriarchy, racism, and discrimination based on social class, religion, or ideology as conflict axes [48,49].

Although Colombia adopted the Convention on the Rights of Persons with Disabilities (CRPD) in 2011, disability has been scarcely addressed in the peace agreement and subsequent documents [50]. While this study does not focus on recognized conflict victims, it is important to situate the participants’ reality within this sociopolitical context. Living in a region with complex social issues, partly due to the conflict and other factors, means that numerous disadvantaged population groups exist. Recognizing persons with disabilities as one of the “worst-off” groups for prioritizing attention is challenging, especially priorities after the peace agreement priorities include attending to direct victims of the conflict, which continues with other actors present.

The interviews were conducted in Cali, Colombia’s third most populous city and economic center. Cali is noted for its multiethnic population, sports culture, and as a cultural hub, but also as one of the most violent cities in Colombia [51].

While ATs can potentially expand the capabilities of people with disabilities, analyzing the geographic, social, political, and economic context of potential users during the design phase is crucial. This consideration is particularly important since using ATs could increase security risks for persons with disabilities.

## Methods

### Study Design and Theoretical Framework

This study adopted a qualitative narrative approach framed within a deductive qualitative analysis strategy [52]. The research was grounded in the CA [18] and the DT [21] as its core theoretical frameworks, with a critical theory lens [53] guiding the inductive reflection. This hybrid strategy enabled the exploration of how AT supports or constrains human capabilities and WB among people with visual disabilities in low- and middle-income settings.

### Participants and Data Collection

This study involved 19 voluntary participants. Eligibility criteria included being 18 years or older; residing in Cali, Colombia, or nearby municipalities; and being blind or visually impaired, without additional hearing, motor, or cognitive disabilities. Eligibility was verified using the Washington Group Questions on Disability Statistics [54]. Purposive and snowball sampling strategies were used. Semistructured interviews (60‐105 minutes) captured life narratives and good life aspirations. A follow-up focus group (n=5) explored their relationship with technology in greater depth, addressing (1) commonly used technologies, (2) perceived utility, (3) opportunities created, (4) desired opportunities, (5) risks, and (6) features enhancing WB.

To ensure rigor, the study used triangulation through a focus group, member checking with the participants, peer debriefings, and a detailed audit trail. Trustworthiness was further supported by verbatim quotes in the findings, which preserved participant voice and supported transparency.

### Data Analysis

#### Overview

Data were analyzed using deductive qualitative analysis guided by the CA. A preliminary deductive codebook was developed based on Nussbaum 10 central capabilities, supplemented with key CA constructs, including resources, conversion factors (personal, social, and structural), agency, and achieved functionings.

Once these were applied, a second layer of deductive analysis was conducted using DT, focusing on identifying insecure functionings, corrosive disadvantages, and fertile functionings.

In addition to the 2-phase deductive stage, an inductive thematic analysis was performed to identify emergent themes not captured by the theoretical framework. This inductive analysis was critically informed by critical theory, allowing interpretation of power asymmetries in technology design, structural exclusion, and user agency.

#### Step-by-Step Coding Procedure

##### Familiarization

The research team read all transcripts multiple times, documenting initial impressions and emotional, social, and technological cues.

##### Deductive Coding: Phase 1 (CA)

Using Nussbaum capabilities, data were categorized into capability domains and analyzed with subcodes for functionings, resources, and conversion factors.

##### Deductive Coding: Phase 2 (DT)

Narratives were recoded to assess the security and fertility of functionings and to identify corrosive disadvantages (eg, stigma and economic precarity).

##### Inductive Coding

Emergent codes such as desire to appear attractive, resistance to confrontation, and wish to explore movement were documented, grouped, and interpreted using critical theory.

##### Integration

Inductive and deductive codes were reconciled through iterative discussions, using analytic memos. For instance, fear of confrontation was related to insecure functionings under “Control over one’s environment,” while vanity versus disability was interpreted within “Affiliation.”

##### Synthesis

Final interpretation focused on how AT shaped each participant’s capability set, aspirations, and obstacles to achieving the good life.

Disagreements in coding were addressed through iterative team discussions until consensus was reached. A reflexivity journal was maintained by the lead researchers to document positionality, epistemic tensions, and interpretive decisions. All coding was conducted manually without the use of software for qualitative analysis. Transcripts were originally in Spanish, and coding was performed in Spanish by native speakers. English translations were collaboratively developed to preserve semantic and cultural accuracy.

### Codebook Summary

Deductive and inductive codes are presented for illustrative purposes (Tables 1-3).

**Table 1.** Deductive codebook (capability approach).

Capability	Subcapability	Code	Definition	Conversion factor type	Example quote
Bodily integrity	Bodily sovereignty	Privacy respect	Situations of physical or decisional invasion	Social	“...they touch you… that’s not necessary. So, it’s like they don’t respect your bodily integrity...”
Bodily integrity	Mobility	Inaccessible transport	Lack of access to safe, navigable transport	Structural	“...the audio announcements don’t work... so how are we supposed to know if the bus is the one we’re waiting for?”
Affiliation	Dignity in service delivery	Discrimination	Being rejected or underestimated during services provision	Social	“...people assume I’m there to beg or ask for something.”
Control over one’s environment	Material control	Institutional barriers	Bureaucratic or policy-related access issues	Structural	“ATMs don’t talk... it’s both accessibility and safety.”

**Table 2.** Deductive codebook (disadvantage theory).

DT^a^ code	Definition	Inclusion criteria	Example quote
Insecure functioning	A functioning being achieved but threatened	Describes fragile access to opportunities	“I only go to the bank with someone I trust. ATMs are not accessible and it’s unsafe.”
Corrosive disadvantage	A single disadvantage that creates multiple deprivations	Barriers that multiply exclusions (eg, inaccessible transport affects work and autonomy)	“If I can’t use public transport, I can’t go to training, or to work. Everything falls apart.”
Fertile functioning	A capability that enables many others	Mentions of tech or contexts that expand autonomy	“My cell phone is essential in my life. It helps me navigate, communicate with others, check when the bus is coming, know where to get off, and get any place.”

a DT: disadvantage theory.

**Table 3.** Integration of inductive themes with deductive frameworks (CA^a^ and DT^b^).

Inductive theme	Definition	Example (quote)	Related capability (CA)	Associated concept (DT)	Interpretive notes
Wish to appear attractive	The desire to look good and express personal style, challenging stereotypes of dependency.	“I consider myself vain despite my disability... so, I would buy more clothes... I would dye my hair...."	Affiliation and bodily integrity	Insecure functioning or symbolic disadvantage	Challenges cultural narratives of disability as asexual or unattractive; signals agency in self-image.

a CA: capability approach.

b DT: disadvantage theory.

### Reflexivity and Strategies for Rigor

Reflexivity was maintained via journals kept by both main researchers. These captured assumptions, affective responses, and positionality reflections. Disagreements in coding were addressed through iterative team discussions and memoing. Credibility was supported through member checking and triangulation using interview and focus group data. Saturation was considered achieved after 19 participants, as no new themes emerged during the final interviews and confirmatory focus group.

### Researcher-Participant Relationships and Empowerment Strategies

Although this study did not follow a formal participatory design framework, several intentional strategies were used to promote ethical engagement and participant empowerment. Informed consent was provided in accessible formats, including Braille and audio, with participants choosing the version that best suited them. Potential interview locations were identified by the research team and evaluated in consultation with key participants, allowing for informed, context-sensitive decisions.

Participants were clearly informed of the study’s aims and the distinct roles of the research teams. Notably, the team responsible for data collection was not involved in AT design, helping to manage expectations and avoid instrumental relationships. Interviews were guided by an ethic of care, privileging participant-led narratives and emphasizing trust, autonomy, and open dialogue.

We also approached participation critically. While participatory methods are often promoted in inclusive design, they may reinforce power asymmetries if not carefully implemented. As Cooke and Kothari [55,56] warn, such approaches can result in a “tyranny of participation,” serving dominant interests rather than those of marginalized groups. To avoid this, our study prioritized epistemic justice by centering participants’ lived experiences as ends in themselves, not as inputs to a predefined design process. Member checking and a focus group enabled deeper reflection and participatory analysis, contributing to more equitable and meaningful knowledge production in line with the ethical commitments of the CA [17].

### Ethical Considerations

The research protocol was approved by the Swiss Federal Institute of Technology Lausanne on research involving humans (056‐2021 and 068‐2022), the Human Ethics Committee of Universidad del Valle (008‐022), Hospital Universitario del Valle (029‐2022), and Instituto para Niños Ciegos y Sordos del Valle (CEI-2022‐02), ensuring fully informed consent and participant anonymity. In accordance with ethical guidelines for research involving human participants, compensation was provided in a manner that did not constitute undue inducement. Participants received reimbursement for transportation costs and were offered food and beverages during data collection sessions. Additionally, they received a modest stipend of approximately 40,000 COP (equivalent to less than US $10) as compensation for their time and participation.

## Results

### Overview

This section presents the main findings of the study, starting with a general description of participant demographics (Table 4) to provide contextual background. The results are then organized into 2 main areas: the first presents the outcomes of the deductive analysis guided by the CA, structured under the subheadings “Notions of Well-Being and Autonomy,” “WB and the Good Life: Common Functionings Identified,” and “Lived Experiences through the Lens of Capabilities and Technology.” The second area includes the inductive themes that emerged beyond the predefined theoretical framework. Although the results of the secondary deductive analysis informed by DT—building upon the initial CA coding—are not included in this section, they are presented in the discussion, where they more effectively enrich the interpretation of structural constraints, genuine opportunities, and the security of valued functionings within participants’ lived realities.

**Table 4.** Participant demographics.

Participant characteristics	Overall
Age (years), mean (SD)	31.1 (10.9)
Sex, n (%)	
Female	7 (36.8)
Male	12 (63.2)
Marital status, n (%)	
Single	13 (68.4)
Common law	3 (15.8)
Married	3 (15.8)
Education, n (%)	
No formal education	1 (5.3)
Primary	1 (5.3)
Completed high school	6 (31.6)
College or university in process	5 (26.3)
Completed college or university	6 (31.6)
Employment status	
Employed	6 (31.6)
Unemployed	6 (31.6)
Self-employed	2 (10.6)
Student	5 (26.3)
Origin	
Urban	17 (89.4)
Rural	2 (10.6)
Self-reported ethnicity	
Afro-Colombian	2 (10.6)
None	17 (89.4)

### Notions of WB

In exploring what constitutes WB for the participants, we inquired about their ideal life and contrasted this with their current experiences. It quickly became clear that disability significantly influences their conception of an ideal life. A couple of participants explicitly noted that their vision of an ideal life had to be “edited” due to their disability, such as stating “my ideal life now that I am blind” or recognizing that their disability constrains their aspirations.

### Autonomy and WB

Participants identified autonomy as central to their WB, emphasizing the importance of freedom in decision-making and the ability to make meaningful choices. One participant articulated this need for autonomy as follows:

A more assertive life would be one in which people first see me as an equal, rather than focusing on my condition. It would be a life where I can make all my own decisions, where having a disability does not limit my opportunities.

[Male, 45 years]

The emphasis on autonomy extends to living without the constraints of disability-related prejudice and ensuring social acceptance. However, the frustration with societal limitations often leads to a pragmatic view of WB, focusing on achievable goals within their control and avoiding, in the process, being assisted by others. For example, a 29-year-old participant described her ideal life as

To have as much autonomy as possible—being confident to go out independently, having a stable job that meets my needs, maintaining good health, and practicing my sport. Total autonomy in my life is what I strive for.

[Participant, 29 years]

### WB and the Good Life: Common Functionings Identified

Work is frequently cited as a critical element of WB. It is valued both for providing financial security and as a source of personal fulfillment. Achieving financial stability and having a fulfilling job are integral to their idea of a desirable life. As one participant who has several part-time jobs with limited hours a week, put it,

I have a job and I am grateful, but I would like to improve my working conditions to better provide for my family and ensure our own home.

[Female, 28 years]

Ownership of a home is another significant aspiration, often linked to job stability. Other desired functionings include pursuing education, maintaining good health, and excelling in sports. These aspirations reflect a broader understanding of WB that includes personal and professional fulfillment.

Moreover, family stability, emotional support, and social connections are also important components of WB for participants.

When one participant (male, 40 years) compared his current life with his ideals, he became aware that he had achieved much of what he considers a flourishing life. He reflected on his accomplishments with satisfaction while acknowledging areas for improvement. Despite this, he emphasized that his experience is not universal among persons with disabilities and that the limitations imposed by disability affect what can be achieved.

At this point, as a person with a visual disability, I believe I have achieved a great deal, despite the challenges. I see others who, unfortunately, lack employment, family, or social connections, or who haven’t been able to fully enjoy life. For me, job stability would make my life 100% ideal. I feel fulfilled because I’ve experienced love, marriage and the journey of being a parent. I have also traveled, engaged socially, and even gone bungee jumping.

[Male, 40 years]

Though the desire for family formation or partnership is less commonly mentioned by those who were single at the time of the interview.

### Lived Experiences Through the Lens of Capabilities and Technology

In examining Nussbaum's central capabilities in the context of individuals with visual disabilities, the analysis reveals both opportunities and barriers that impact their daily lives.

#### Life

This capability is initially affected by the sociopolitical context of the country and specifically by the social and infrastructural conditions of the city. As one participant recounted,

We left Cauca, displaced by the violence. We also lived in Putumayo for a few years, but in 2009 they killed my brother, and we had to leave again—displaced by the violence.

[Female 21 years]

(Cauca and Putumayo are departments in southern Colombia that have been heavily affected by armed conflict.)

In some cases, participants explicitly linked their visual impairment to episodes of violence. One participant explained:

I’ve been living in Cali for four years, which is also how long I’ve had this condition (low vision). I was living in my village at the time, and I came here to study. It was around then that the incident happened — someone attacked me to steal my phone and other belongings.

[Male, 23 years]

Similarly, another participant shared:

I lost my sight when I was 20 years old, as a result of a firearm injury. After that—well, thank God—I recovered well after about a year. It was like a sabbatical year, where I didn’t really do anything, because at the time, I didn’t know how to get my life back on track.

[Male, 29 years]

Beyond the direct consequences of violence, participants also pointed to everyday urban dynamics that fail to prioritize the safety and dignity of people with visual disabilities. In particular, unsafe social behavior—such as the frequent disregard for traffic rules by drivers—emerged as a recurring concern. Falls, collisions, and traffic accidents were identified as major threats to personal safety and mobility. Several participants described incidents in which they had sustained serious injuries.

I fell into the gap and ended up on the road, between the station platform and the bus.

[Female, 31 years]

One participant told during the interview that her brother, who was also blind, was the fatal victim of a traffic accident 2 months earlier, when he was run over by a car while crossing the street.

#### Bodily Health

Participants have access to the Colombian health system and benefit from rehabilitation services that focus on orientation and mobility. Participants from rural areas only accessed these services once they arrived in Cali. Regarding access to basic AT, although there is a route to obtain it through the health system, a couple of participants reported that it is cumbersome. However, they say that canes are more readily available through the mayor’s office.

Financial instability has led to periods where participants’ nutrition and living conditions have been compromised. As the following quotes illustrate:

When I was little and still living with my mom and siblings, we got evicted because we couldn’t pay the rent anymore. We ended up moving into the first place we found, but it was full of rats—and I’m really scared of them.

[Female, 54 years]

There were hard times, times of real scarcity. Sometimes you had to really stretch things—like cutting back a lot on food—just to pay for basic services, because skipping those payments wasn’t an option.

[Female, 21 years]

#### Bodily Integrity

Participants have received training that enables some degree of autonomy in navigating the city using a white cane. Nonetheless, the freedom to move around safely is frequently compromised by risks of physical injury and insecurity.

Technology plays an essential role in facilitating mobility for participants, with apps aiding navigation and transportation. However, while most participants own smartphones, many do not have consistent access to mobile data to ensure these apps function effectively.

Some participants limit navigation to familiar areas or rely on human assistance due to safety concerns and technological limitations. Experiences of mugging and other safety risks further hinder their mobility, independence, and willingness to use technology in public.

My phone was stolen at the station while I used a transportation app to check my bus arrival time for work. It was early, and I thought I was alone since I heard no one nearby.

[Male, 40 years]

Others are not unaware of the risks in public spaces—they just have a lower perception of the danger, or they choose to manage the risk rather than give up the technology altogether. Instead of avoiding it, they take precautions to reduce the chance of getting robbed.

I’m one of those people who says that blind folks don’t get robbed — and yeah, people laugh when I say that, hahaha. Sure, I know plenty of blind people who’ve been robbed — but that’s because they gave papaya (in Colombia, that means making yourself an easy target). You just can’t give papaya! I’ve been in dangerous situations before, but thank God, I’ve always gotten out safely. I’ve never been robbed.

[Male, 39 years]

During the focus group, when participants discussed the disadvantages or risks of using technology, the first issue that came up was personal safety. As illustrated in the following quote:

The first thing that comes to mind is safety. For example, around here everyone talks about Lazarillo and all those apps—but I don’t use any of them, because I don’t take my phone out. We become very visible when we pull out our phones, hold them to our ear, or start using them. That makes me an easy target for someone to steal it. Given the context we live in, it just doesn’t work for me—it actually makes me feel even more unsafe.

[Male, 37 years]

After sharing stories about people they knew who had been victims of phone theft, participants went on to describe the strategies they use to avoid becoming targets themselves. For example, the 2 female participants said they use headphones so they do not have to take their phones out of their pockets, and they hide the headphones with their hair.

Safe mobility is essential for achieving various functionings, but its impact extends beyond simply moving freely and avoiding aggression. One participant noted that communication is a major challenge for those unable to move independently. He added that autonomous mobility is crucial for meaningful interactions, including forming personal relationships, such as finding a partner.

Not walking alone makes others, even friends, perceive a person with a disability differently. They already have questions about your disability, which you must address when interacting with them. However, if they see you always relying on someone, it becomes much harder to connect with them.

[Male, 24 years]

Several interviewees noted that being a woman heightens their fear during independent travel, whether on public transport or walking in the streets. One participant shared,

Regardless of disability, ethnicity, or economic status, being a woman increases the risk of aggression, especially sexual violence. Being a woman in Cali, and in the areas I frequent, is terrifying. People often mock me with idiotic comments. Just the other day, someone said, ‘Oh, you’re so pretty, but what a pity about that stick (the cane).

[Female, 21 years]

#### Senses, Imagination, and Thought

With the exception of 2, all participants completed high school, and about half (n=10) pursued higher education. AT, such as screen readers and text-to-speech apps, are widely used. However, those who studied before advanced technologies faced significant barriers, such as manually converting documents to audio or advocating for accommodations. Despite these challenges, most described their education positively but noted limited career options due to inaccessible educational and professional pathways. As a 19-year-old participant explained,

I chose law by default. I picked it because I’m good at public speaking, but do I truly enjoy it? No. Our options are limited, even if they seem abundant. While barriers can be overcome, I’m not willing to exhaust myself doing so.

[Participant, 19 years]

#### Practical Reason

Participants’ decision-making reflects strong priorities and resource management, aided by technology for accessing information and connecting with advocacy groups. However, uncertainty and anxiety about the future, especially among those newly adjusting to disability, hinder long-term planning. As one 19-year-old male shared:

I don’t think about the future. It doesn’t exist—it’s just conjecture, you know? I’ve learned not to stress over it. For me, the future would mean a stable job, continued training, and… that’s it.

[Male, 19 years]

While technology plays a vital role in accessing information and social networks, accessibility issues remain a significant challenge.

#### Affiliation

People mostly connect with family members and others with visual impairments, but they find it hard to build relationships beyond those close groups. Strong support networks often form in educational or social contexts, such as public libraries, and through cultural or adapted sports activities. However, barriers persist, including discrimination, ableist remarks, and inaccessible public infrastructure, which often prioritizes property over safety.

Do you think people respect the few podotactile guides that exist? No, they park their cars on them. If you get too close, they say, "You're going to scratch my car," forcing you onto the road and into danger.

[Female, 54 years]

Participants also face employment challenges, frequently working in roles they are overqualified for or in part-time positions with insufficient pay. Visual impairment complicates social interactions, as one participant noted:

Sometimes relationships begin with a look, but without that, it’s already one point less, isn't it? And then there’s the hesitation people feel toward disabilities.

[Male, 45 years]

Accessing services adds to these difficulties. City buses equipped with loudspeakers frequently have these systems turned off, while shopping malls present physical accessibility barriers; additionally, salespeople often fail to recognize persons with disabilities as customers.

I’ve never gone shopping alone. I have no way to orient myself in a mall, and I don’t like arriving somewhere unfamiliar and having to wait for someone to help me. Salespeople usually don’t realize that a blind person with a cane can be a customer.

[Female, 31 years]

While technology, such as social networks and apps, has improved accessibility, significant issues remain, such as inaccessible ATMs, banking apps, and dating platforms such as Tinder (Match Group, Inc), where user photos lack proper descriptions.

#### Other Species

Participants express satisfaction with their ability to engage with nature through their other senses. Despite some limitations, they continue to find joy and fulfillment in their sensory experiences and interactions with the natural world.

#### Play

Available opportunities include participation in adapted sports and recreational activities supported by local initiatives. Participants enjoy activities such as listening to music, radio programs, and audio-described films. However, financial limitations restrict their access to a broader array of recreational options.

#### Control Over One’s Environment

Political involvement is marked by participation in advocacy activities and voting, though challenges such as discriminatory treatment and the need to navigate physical barriers persist.

Material control is constrained by the scarcity of full-time employment and inadequate financial stability. Issues with banking and consumer services, compounded by inaccessibility and discrimination, affect their ability to manage material resources effectively.

#### Themes Identified Through the Inductive Analysis

The inductive analysis of interviews and focus group discussions revealed 3 interrelated themes that reflect participants’ subjective experiences and priorities in everyday life. These themes include the aspiration to express and expand their movement potential, the importance placed on maintaining a positive and attractive personal appearance, and the strategies used to avoid confrontation and maintain social harmony.

#### Desire to Exploit Movement Potential

A prominent theme in the interviews was the frustration many participants felt regarding their limited mobility. They expressed this frustration over their inability to fully experience freedom of movement, navigate the city and its spaces because of infrastructural barriers, and socialize due to a lack of bodily experience. Additionally, they cited deficiencies in ADs as a contributing factor.

Participants frequently noted that the cane, while essential, places limitations on their freedom of movement and enforces a rigid posture, making their gait different from others.

The cane forces you to maintain a fixed posture, which limits your ability to move naturally. Unlike others who can easily turn and walk freely, we can't move the same way.

[Male, 47 years]

Many expressed a strong desire to engage in motor activities that are currently restricted for them, such as riding a bicycle, running, swimming, dancing, and skating. There was a clear enthusiasm for speed.

I long to run, but to run alone. I yearn to walk freely, without a cane, moving as I please. I would love to ride a bicycle.

[Male, 47 years]

In the realm of dance, beyond the desire for self-expression through movement and the joy of dancing itself, this activity serves as a crucial means of socialization—especially in this city, renowned as the salsa capital.

Well, I like to dance but I don't know how to do it well. I love dancing. ...I do it my own way, in a clumsy way, but I do it. ... Yet, I would love to master dancing.

[Female, 56 years]

In terms of technology, while some participants acknowledged the crucial role of the cane, they expressed a need for an AD that would enhance their mobility with greater security and fluidity, without compromising their sense of independence or human qualities. Additionally, they emphasized the importance of keeping at least one hand free, as the other one is currently occupied by the cane.

Although many express hope and enthusiasm for the potential of AT in enhancing mobility, others caution that the technology may not address the factors that limit their ability to navigate the city, particularly those related to human interaction. As one participant shared in the following quote:

Many variables are involved. The recklessness of drivers is one of them. I've heard that in other countries, people really respect traffic signs and regulations. If that were the case here, one could cross the street when the light is red, because you could trust that drivers would obey the rules. But that’s not the case here. Traffic lights are often ignored. Motorcycles also present a significant issue. Even when there’s a pedestrian crossing, not all vehicles line up properly. If they did, I would know the boundaries of the space I need to cross. Unfortunately, that doesn't happen. When I'm crossing at a traffic light, I may plan to pass on one side, but I find one car ahead of another, and then a motorcycle suddenly appears.

[Male, 40 years]

#### Desire to Look Attractive

The theme of looking attractive surfaced notably, particularly among female participants. One participant (female, 29 years) expressed frustration with online shopping due to insufficient garment descriptions. She also desired AD that would enable her to perform personal grooming tasks independently, such as painting her nails and plucking her eyebrows, activities she used to do on her own before acquiring a visual disability.

Another participant (female, 27 years) mentioned that if she had the financial means, she would purchase more clothes and enjoy activities such as hair dyeing and makeovers. Regarding mobility AT, she expressed a desire for a discreet or stylish device that could detect high obstacles and match her clothing. She confessed, somewhat apologetically, “I´m somewhat vain, despite my disability.”

A 21-year-old female participant noted her reluctance to use the cane when she had the option of human assistance, explaining that the cane did not complement her style.

I think it’s more like this: it’s not as if I say, 'Oh, that cane matches my style, how exciting, man!' No, it’s not like people come up and say, 'What a cool cane, it goes great with your shirt.

[Female, 21 years]

The desire to appear attractive also extends to social and romantic interactions. Participants revealed that concerns about attractiveness influenced their willingness to use certain AT. For example, one participant (male, 40 years) described how societal norms and personal shame affected his acceptance of using a cane.

Even though I knew how to get around with my cane, I always relied on my grandmother, who met me at the bus stop every day. One morning, I couldn’t let her know I’d be early, so I ended up waiting three hours instead of using my cane. That experience made me realize I needed to start using it. Honestly, the shame I felt was mostly about what the opposite sex might think of me. I guess those feelings are pretty common for young people, shaped by societal pressures and insecurities.

[Male, 40 years]

A female participant (57 years old) noted that her daughter’s initial embarrassment about using a cane was linked to teenage concerns about appearance:

She felt embarrassed to use the cane, especially because, as girls start to become aware of their attractiveness to boys, they may feel self-conscious. However, she eventually came to accept it.

[Female, 57 years]

Another female participant noted that her embarrassment about using the cane is heightened when she is around peers of her age and the opposite sex who do not have disabilities. She mentioned that she has rarely discussed this issue, as she recently recognized her feelings of shame and struggled to openly acknowledge them.

One male participant (45 years old) described his experience after trying a prototype of an AD that included glasses equipped with an obstacle-detection camera, expressing strong reservations.

I wouldn't even go out in public wearing those. First, they pose a danger—I could get robbed. Second, they look terrible. If you wore them to a party, do you really think anyone would find you attractive?

[Male, 45 years]

#### The Bid to Avoid Confrontation

The theme of avoiding confrontation appears in various contexts throughout the study. For instance, some participants avoid expressing their political views to prevent arguments or physical confrontation.

One participant noted that while he is open about his political convictions, he often faces derogatory remarks about his disability when others disagree with his views.

The desire to avoid confrontation also emerges when participants describe their experiences with ableism. Many report feeling frustrated by ableist treatment but choose to remain silent to avoid conflict. For example, a participant shared an experience:

They see you with a cane and automatically lock you in the world of the blind, of the <what a pity>, of <how do you manage to walk by yourself?>. Also, they think they have the right to ask questions and make silly expressions. One day I was with my cane, I sat in the Bus and a woman came up behind my ear and said to me: <You have to pray a lot so that the spirit of blindness leaves your life>

[Female, 29 years]

Such incidents often deter individuals from using ADs, fearing further stigma.

Another participant recounted an incident where he felt threatened after unintentionally pushing another man while stumbling in a public bathroom. Since he was in a familiar location, he was not using his cane. To prevent a confrontation, he apologized and explained his condition.

This theme underscores the dual role of the cane. While it can signal vulnerability and deter aggression, it can also trigger stigmatization. Participants sometimes avoid using the cane to prevent negative reactions, which may impact their overall functionality and social participation.

## Discussion

### Principal Findings

The discussion begins by highlighting the centrality of autonomy in participants’ conceptions of WB and its implications for AT design (“The Good Life and WB”). It then reorganizes functionings into 3 analytical categories: “Most Valued Functionings,” “Insecure Functionings,” and—based on their enabling or limiting effects—“Corrosive Disadvantages” and “Declustering Disadvantage.” This is followed by a review of participants’ experiences with specific technologies in comparison to existing literature (“AT and the Creation of Opportunities and Barriers in Participants’ Daily Lives”). The final sections present emergent sociocultural themes (“Themes Identified Through the Inductive Analysis and AT”) and integrate all theoretical strands into a situated analysis of agency, risk, and design (“AT Between Opportunity and Constraint”).

### Main Results

#### The Good Life and WB

This study aimed to examine how AT creates opportunities and barriers in participants’ daily lives and influences their WB. We first explored the concept of an ideal life, which informed our understanding of participants’ perceptions of a good life and WB.

This valued way of life includes, but is not limited to, WB [57]. WB, on the other hand, is assessed in terms of the achievement of functionings that people value and incorporate into their conception of a good life [34].

While interviewees widely valued agency, opinions on autonomy varied. A few participants preferred autonomy over independence, noting that dependence is a natural part of life. However, others viewed autonomy as minimizing reliance on others for daily activities.

This distinction is especially important when considering how AT is developed for persons with disabilities. While the desire for independence is a valid and often central driver in AT design, it can sometimes overshadow other dimensions of daily life. Bennett et al [58] argue for reframing AT through a lens of interdependence, emphasizing that technologies are embedded in relationships, routines, and environments—not isolated solutions. Similarly, Joskow et al [59], using the CA, show that the value of AT lies not only in enabling functional tasks but also in supporting participation, emotional WB, and meaningful choice. These perspectives highlight the need to design AT with a more holistic understanding of how persons with disabilities navigate and shape their worlds.

This becomes particularly evident when comparing the experiences of different users. One male participant, reflecting on the challenge of crossing at traffic lights, shared, “An AT device wouldn’t work as well for that kind of situation, because it’s still dangerous.” Even with a guide dog, he noted, it’s hard to manage the risks posed by reckless drivers. He also described the value of informal social interactions that occur in public space, “Sometimes I walk a bit on my own and someone will say, ‘Hey, where are you headed?’...I’ve even met people that way—people who later became friends.”

In contrast, a female participant expressed a clear preference for technological assistance over human help, “I would much rather have a device than a person helping me, because I’d feel like I’m working together with the device—and that makes me feel more autonomous...it’s perfect!” She added, “Regardless of its size or how it looks, having a tool that helps us move around independently is incredibly important.” Even if the device is “big, ugly, or heavy,” she saw it as empowering—what mattered most was the sense of self-reliance it enabled.

Both perspectives offer valuable insights for AT design. Listening to this diversity of lived experience helps avoid defaulting to a one-size-fits-all model centered solely on individual autonomy. While preferences like those of the second participant—who expressed enthusiasm for any device that enhances independence regardless of size or appearance—may seem appealing to designers because they reduce complexity, relying solely on such responses can be misleading. If designers prioritize these simplified preferences without accounting for broader user expectations—such as comfort, discretion, aesthetics, or social dynamics—they risk developing yet another device that ultimately fails to meet the needs of a wider group of potential users. Truly inclusive design must engage with this heterogeneity, acknowledging not only aspirations for autonomy but also the embodied, emotional, and relational dimensions of navigating everyday life.

#### Most Valued Functionings

Among the functionings most valued by participants for a good life, material security—primarily through work as a source of income—stood out. Other key functions included affiliation, education, sports, health, and mobility. This aligns with Wolff and De-Shalit findings, which identify core categories of functioning, including life, bodily health, bodily integrity, affiliation, environmental control, and senses, imagination, and thought [21].

#### Insecure Valued Functionings

Although these functionings are highly valued by participants, they remain insecure for a significant proportion of them. Employment challenges are prevalent, reflecting findings from other studies that report low employability, poor working conditions, lower wages, and job instability for people who are blind or have low vision [60-63].

Similarly, while participants emphasize the importance of family and social networks, they also highlight barriers to full inclusion and social participation, making affiliation another insecure functioning. These challenges are consistent with existing literature, which associates limited social engagement with reduced WB and identifies significant social stigma and restricted access to goods and services for persons with visual disabilities, in both public and private spaces [62,64-67].

Given the violence, physical barriers in urban areas, and societal attitudes, life and bodily integrity also emerge as insecure functionings. Empirical studies support these findings, citing falls, life-threatening injuries, and higher mortality risks among persons with visual disabilities [68,69].

Education, including higher education and vocational training, is generally seen as a secure functioning. Despite Colombia’s high illiteracy rates among persons with disabilities, participants in this study appear to have attained higher levels of education than the national average for persons with disabilities [70,71].

Finally, although all participants have health insurance, some have faced precarious situations that question health’s security as a functioning, particularly given the material insecurity caused by unemployment.

#### Corrosive Disadvantages

##### Overview

Efforts for social justice should prioritize groups experiencing multiple disadvantages. Participants highlighted unemployment, lack of affiliation, and mobility barriers as significant corrosive disadvantages.

Unemployment or underemployment threatens basic functionings such as housing, food, health, and life, while also limiting leisure, education, and recreation due to financial constraints. Studies show that income loss exacerbates social isolation, diminishes status, and is linked to reduced WB, limited access to healthcare and education, declines in health, and increased mortality risk [72-75].

Insecurity in affiliation similarly undermines other functionings. Participants often face disrespect and unequal treatment, which compromise job security, environmental control, and freedom of movement without risking safety and WB. Research links social exclusion with limited participation in economic, social, and cultural life, resulting in poverty, unemployment, inadequate health care, and poor housing for persons with disabilities [67,76-78].

Restricted mobility constitutes another corrosive disadvantage, as it directly undermines functionings such as access to essential services, physical activity, communication, WB, and social inclusion. Research highlights the importance of sensorimotor abilities in social interaction [79,80], with decreased mobility linked to isolation and lower satisfaction [81].

##### Declustering Disadvantage

Identifying corrosive disadvantages highlights the importance of fostering fertile functionings—those that support resilience and WB. Participation in adaptive sports is a clear instance, as research shows that each additional year in adaptive sport is associated with a 4% increase in employment likelihood among people with physical disabilities [82]. In our study, involvement in sports provided income, travel subsidies, and social connections. It supported capabilities such as affiliation, bodily integrity, and control over one’s environment.

Employment also served as a fertile functioning when conditions were fair, remuneration equitable, and respect for dignity was present. Studies indicate that AT access in the workplace is strongly linked to employment retention and productivity among individuals with visual disabilities [83].

As pivotal conversion factors, AT tools such as screen readers and accessible mobile apps enabled participants to use digital platforms daily—facilitating email, social media, and work tasks [84]. As a result, these technologies not only support function, but also reinforce confidence and autonomy.

Although participants used diverse ATs, this paper focuses on representative examples. Further research should explore a broader array of technologies and how they foster capability and reduce disadvantage across different daily contexts.

Mobility technologies warrant particular attention. Studies show that interactive navigation tools can significantly enhance users’ spatial confidence and independence [85]. Participants emphasized that AT, which enables seamless environmental interaction—not just orientation or obstacle avoidance—enhances their movement potential, autonomy, self-efficacy, and engagement with public space.

##### AT and the Creation of Opportunities and Barriers in Participants’ Daily Lives

AT has opened multiple avenues for expanding capabilities among persons with visual disabilities, supporting valued functionings across educational, occupational, and social domains. Participants in this study described how information and communication technologies have enhanced their performance in academic tasks, enabled participation in the labor market, and expanded opportunities for leisure and connection. These tools facilitated informed decision-making, strengthened social networks, and increased personal visibility in both professional and interpersonal contexts [86].

Mobility technologies, including white canes and mobile navigation apps, were also perceived as crucial for promoting spatial autonomy. They allowed individuals to traverse their environments more independently, access public and private spaces, and engage in civic and social life. AT also enabled participants to perform essential tasks such as banking and online shopping, thereby enhancing their control over daily routines and improving their sense of independence.

However, participants also identified significant barriers to AT use—barriers that highlight the ambivalence of technology in their daily lives. As Moser [87] argues, technology does not simply empower—it also has the capacity to withdraw agency by rendering difference hypervisible or by reinforcing stigma. This tension was evident in the accounts of participants who, while recognizing the utility of AT, expressed concern about its potential to attract unwanted attention, particularly in contexts marked by high levels of urban violence. In these settings, visibility becomes a liability, increasing the risk of theft or social targeting.

This ambivalence was especially pronounced in public spaces. While some participants preferred discreet technologies that do not mark them as disabled, others acknowledged that visible devices, such as white canes, could help signal their needs and elicit assistance. These findings resonate with Dos Santos et al, who found that aesthetic and symbolic dimensions strongly influence the acceptability and use of AT [88]. Similar insights have emerged in studies from the Global South, such as Barbareschi et al [89] work in Kenya, where ADs can either mitigate or exacerbate stigma depending on the sociocultural context.

Thus, the potential of AT to support WB is conditioned not only by its functional properties but by its alignment with users’ social environments and identities. When well-designed and contextually appropriate, AT can foster fertile functionings—such as autonomy, affiliation, and environmental control. However, when AT is experienced as stigmatizing, risky, or misaligned with local realities, it can become a source of discomfort or even exclusion. This dynamic reinforces the importance of attending to not just technical efficiency, but also social safety, emotional meaning, and contextual sensitivity in design and policy.

##### Themes Identified Through the Inductive Analysis and AT

In Latin America, physical appearance holds significant value, rooted in colonial legacies that entrenched hierarchies by emphasizing differences between colonizers and the colonized. These hierarchies associated power with traits such as being European, patriarchal, White, wealthy, heterosexual, and able-bodied. Disability, conversely, was linked to negative characteristics such as unproductivity and rebellion, devaluing disabled bodies and dissociating them from beauty [90,91]. This detachment was reflected in interviews, where participants either avoided discussing physical appearance or addressed it obliquely. One participant remarked, “I am somewhat vain despite my disability.”

The desire for attractiveness was particularly pronounced among women, aligning with research that connects beauty ideals to women’s roles in Latin American cultures [92,93]. In Colombia and Brazil, beauty is often tied to socioeconomic status and professional opportunities [94,95]. While beauty remains culturally valued, ADs such as canes—symbolizing disability—are perceived as aesthetically unappealing and are sometimes rejected.

Aesthetics influence social interactions beyond economic implications. Participants expressed a desire to appear attractive to the opposite sex but seldom mentioned romantic aspirations, potentially reflecting aspirational frustrations. This tension between appearance and relational expectations emerged more clearly in the focus group discussions, which revealed the need for greater accessibility on dating platforms such as Tinder, highlighting participants’ openness to using AT to facilitate social interactions and romantic relationships.

Limited mobility emerged as a recurring challenge, restricting participants’ navigation skills and engagement with urban environments. Kukla's concept of spatial agency—the ability to autonomously use space—captures this dynamic. City navigation requires interaction with people and environments, relying on cues such as body language, eye contact, and street signs [96]. Existing mobility AT primarily addresses orientation and obstacle detection but lacks tools for effective environmental engagement. Participants’ adaptive strategies highlight the need for improved AT design. Kukla also emphasizes that movement pace and aesthetic norms influence spatial navigation [96]. Participants underscored that AT should enhance autonomy while enabling natural interaction with tasks and people, without disrupting rhythms.

AT design must prioritize the user’s holistic experience, accounting for the diverse environments they navigate [97]. This context extends beyond controlled settings, incorporating cultural meanings of disability, prior interactions, and broader social, historical, and political factors. The social realities of designers in the Global North often differ significantly from those of persons with disabilities or users in the Global South, as do their perceptions of aesthetics and its functional role in daily life.

##### AT Between Opportunity and Constraint: A Situated Analysis of Agency, Risk, and Design

In Colombia, defying dominant social norms often entails serious consequences, frequently enforced through violence. This violence is not limited to state institutions; nonstate actors—including armed groups and criminal organizations—have also enforced normative boundaries. Individuals who challenge socially constructed expectations—such as LGBTIQ+ (lesbian, gay, bisexual, transgender, intersex, and queer) persons, Indigenous peoples, Afro-Colombians, and social leaders—are disproportionately subjected to threats, forced displacement, and even assassination [98,99]. In cities such as Cali, such patterns have at times been reinforced by criminal governance structures that monitored daily life and punished perceived transgressions, exerting coercive control over entire neighborhoods [100]. In these contexts, expressing a nonnormative identity or dissent becomes a high-risk act, revealing how violence operates as a mechanism of civic regulation.

This coercive environment encourages conformity not through democratic consensus but through fear. Individuals internalize the threat of retaliation, adapting their appearance, behavior, and speech to avoid becoming targets [98]. Over time, this normalizes silence and compliance, particularly in communities with limited institutional protection. Violence, therefore, becomes a structuring force that shapes notions of “acceptable” citizenship and sustains systems of exclusion.

Within this broader context, ableism functions as a parallel yet distinct form of disciplinary control. In Colombia, persons with disabilities who defy normative expectations—by asserting rights, demanding visibility, or rejecting passive roles—often face institutional neglect, attitudinal bias, and spatial exclusion. The Constitutional Court has described this as the product of “a segregating and exclusionary culture” that restricts life trajectories for persons with disabilities [101]. In conflict-affected areas, civic engagement can become a risk in itself, as some persons with disabilities avoid visibility out of fear of losing access to essential services [102]. Ableism reinforces conformity not through overt coercion, but through anticipated punishment—governing behavior, silencing dissent, and normalizing invisibility [103].

From the perspective of the CA, these exclusionary and coercive dynamics represent severe constraints on individuals’ real freedoms to pursue lives they have reason to value. Structural violence, institutional neglect, and everyday surveillance undermine central capabilities such as bodily integrity, affiliation, and control over one’s environment [18]. These forces not only restrict access to resources but also impair agency itself, fostering anticipatory compliance and silencing non-normative expression.

While the CA emphasizes agency and meaningful choice, it has been critiqued for undertheorizing the sociopolitical and ideological conditions that suppress agency—particularly in contexts marked by systemic domination and fear [20,104]. Although Sen acknowledges power structures through the notion of conversion factors and has addressed issues such as identity-based violence and gender inequality, he provides only a partial theorization of how social and institutional forces constrain choice [20]. In response, critical theorists emphasize that agency is always situated—shaped by hegemonic ideologies, historical inequalities, and structural constraints [20]. These insights enrich the CA by highlighting how oppression is not only material but also discursive and normative.

AT becomes a key analytical entry point in this discussion. Within the CA, AT functions as both a resource and a conversion factor—potentially expanding real freedoms by enabling valued functionings such as mobility, communication, and social participation [9,18]. Yet in contexts shaped by criminal governance, stigma, or weak institutional presence, AT’s capacity to enhance capabilities may be compromised. Fear of surveillance, ridicule, or exclusion can deter individuals from using AT, effectively transforming it into a corrosive disadvantage—a concept from DT that refers to resources that worsen users’ conditions in hostile environments [21].

In cities such as Cali, where high levels of urban violence intersect with limited protection for vulnerable groups, participants reported avoiding certain technologies out of fear for their personal safety. While navigation apps and mobile-based tools might enhance autonomy in principle, in practice, they often increase visibility and perceived vulnerability in public space. As shown in the findings, such risks lead many to forgo potentially beneficial technologies, not because of a lack of interest or understanding, but because the act of using them carries a social and physical cost that undermines the very freedoms they are meant to support.

This reflects a broader pattern in which structural violence becomes embedded in everyday decision-making. Technology adoption is shaped not only by access and design, but also by how individuals negotiate exposure, safety, and autonomy in hostile or unstable environments. In this sense, the very tools intended to promote inclusion may reinforce exclusion when they are not aligned with the social conditions and lived experiences of their users.

Moreover, AT design and policy often reflect biomedical or market-driven logics, failing to account for cultural preferences, affective experiences, and social risks. Research shows that when AT is perceived as overly medicalized or aesthetically stigmatizing, it is more likely to be abandoned—despite its functional benefits [105,106]. In Latin American contexts, aesthetic appeal and discretion are not merely preferences but protective strategies in public spaces marked by insecurity. Orellano-Colón et al [105] found that Hispanic users preferred AT devices that did not draw attention, valuing discretion and aesthetics alongside functionality. In Colombia, where individuals must navigate public visibility with caution, design decisions can significantly impact the acceptability and sustainability of AT use.

Emotional responses—such as shame, pride, or resignation—often remain unspoken but can be inferred from narrative silences, reflecting adaptive preferences. Rather than ignore these silences, designers should interpret them as meaningful indicators of unmet needs—shaped by structural disadvantage—and take them into account in participatory design.

Given that the aim of this study was to understand the role of technology in the lives of people with visual disabilities in LMICs, and how AT shapes opportunities, barriers, and WB, the findings underscore both the promise and limits of AT. While AT cannot resolve the structural inequalities that underpin marginalization, it can be designed to mitigate their effects. This requires that designers pay careful attention to users’ cultural values, local forms of risk management, and context-specific aspirations. Culturally sensitive design that values emotional WB and aesthetic dignity is more likely to foster meaningful adoption and sustained use.

Applying the DT alongside the CA allows for a more nuanced understanding of how AT impacts people’s lives. It invites researchers and designers to consider not only what a technology enables, but what it may jeopardize, particularly when social or environmental conditions are hostile. As the findings show, AT that is context-blind risks becoming ineffective or even harmful. Design processes must therefore attend to fertile functionings—those that support broader forms of agency and resilience—while actively avoiding the reproduction of corrosive disadvantages.

### Limitations

Although the study used rigorous qualitative methods and achieved thematic saturation, the sample size (N=19) and composition—skewed toward younger and more educated urban residents—may limit the diversity of perspectives captured. Future research should include larger and more demographically diverse samples, especially individuals from rural areas, older adults, and persons with visual disabilities with intersecting marginalized identities, to further explore how AT and structural disadvantage interact in varied sociocultural contexts.

While the study aimed to include participants from both urban and rural areas, the final sample was predominantly urban. This may have limited the representation of geographically diverse experiences.

Additionally, the snowball sampling strategy, while effective in reaching a specific population, may have introduced bias by concentrating perspectives within existing social networks.

Certain themes, such as romantic relationships, were underexplored due to study design limitations. However, the focus group offered deeper insights, showing that group discussions encourage openness on sensitive topics.

Even if this study does not cover all 10 of Sen's capabilities, it supports the framework’s relevance in analyzing AT users’ needs. Future research should further explore the specific characteristics of AT and how they align with users’ daily realities.

### Conclusions

This study sheds light on how AT is woven into the everyday lives of people with visual disabilities in Colombia—not just as tools, but as companions, barriers, enablers, and sometimes sources of tension or silence. In a context marked by inequality, insecurity, and deep social stigma, AT does not operate in a vacuum. Whether it supports or hinders WB depends on how it fits into people’s lived realities, relationships, environments, and desires.

By combining the CA with DT and a critical lens, we were able to see not only the functionings participants valued—such as education, work, and independence—but also the emotional and social costs of trying to pursue them. Technologies that might promise autonomy in one context could increase vulnerability in another. A navigation app or white cane, for instance, might enhance freedom, but also attract unwanted attention in unsafe public spaces. For many, using AT meant balancing aspirations with risk.

Participants told stories of resilience, but also frustration. They spoke of wanting to be seen as attractive, of yearning for movement without fear, of choosing silence over confrontation to stay safe. Others left things unsaid—particularly around relationships and intimacy—pointing to desires that are often overlooked, even by well-intentioned designers and researchers.

These findings remind us that the voices of people with disabilities in the Global South need to be at the center of technology design—not just in consultations or interviews, but as full collaborators in research and development. The choices they make, the things they value, and even what they choose not to say are powerful forms of knowledge. Listening deeply to these experiences helps us understand what truly makes a technology meaningful.

To create technologies that genuinely support WB, we must look beyond technical fixes. We must ask, does this technology help someone live the life they have reason to value? Does it support their dignity, their safety, their hopes? These questions cannot be answered by engineers or policymakers alone. They require interdisciplinary collaboration—between designers, social scientists, humanists, and most importantly, people with disabilities themselves.

Ultimately, this study shows that AT holds enormous promise—but only if it is rooted in empathy, shaped by real-world complexity, and open to the diverse ways in which people navigate, resist, and imagine their worlds.

## Abbreviations

AD: assistive device
AT: assistive technology
CA: capability approach
CRPD: Convention on the Rights of Persons with Disabilities
DT: disadvantage theory
FARC-EP: Revolutionary Armed Forces of Colombia
LGBTIQ+: lesbian, gay, bisexual, transgender, intersex, and queer
LMIC: low- and middle-income country
WB: well-being
WHO: World Health Organization
